# Case of Pleuritis

**Published:** 1819-02

**Authors:** Richard Lanyon

**Affiliations:** Licentiate of the Society of Apothecaries, London, &c.


*For the London Medical and Physical Journal.*

*Case of Pleuritis;* by RICHARD LANYON, Jun. M.R.C.S. Licentiate of the Society of Apothecaries, London, &c.

RICHARD REED, the subject of the following case, is a man about 30 years of age, who, by over-fatigue and industry, has very much impaired his constitution; so that the *stamina*, which previously manifested the vigor of youth, now bore evident marks of rapid decay. Thus disposed to disease, he was, on the 28th of last month, seized with a pain in his side, about the sixth rib; and, after a while, the left was similarly affected: the pain, however, was not to any violent degree. When I saw him, he complained also of pain in the region of the sinciput. Pulse soft and natural; slight cough, with a load on his chest, unattended by any marked symptom of fever. With a view of relieving the pain in the head, and restoring the balance to the circulation, the following mixture was ordered:—℞. Tincturæ digitalis, ♏. xxx. Syrupi simplicis, f. ʒvj. Aquæ Puræ, f. ℥v. M. ft. mistura, cujus capiat æger cochlearia duo sextis horis. A simple aperient, with sulphate of magnesia, was also enjoined.

29. A.M.—Had little or no sleep last night, notwithstanding the pain in the head is much alleviated. Pain in the left side more severe than in the right, and is more distressing than yesterday. Pulse not varying from the natural standard. The aperient was effectual in its operation.—Mistura ut heri repetenda.

30. A. M.—Pain in both sides augmented; slept well during the greater part of last night; bowels sufficiently lax. A blister was ordered to each side. Sixteen ounces of blood were drawn from the arm, which did not exhibit the usual characteristics of inflammation.

December 1st.—Slept well at intervals, though profuse perspiration came on while asleep. The pain suffers no mitigation, but is now felt more sensibly above the situation of the blister on the left side; the pain on the right side being much relieved. All the leeches which could be procured were applied, amounting, however, only to the small number of five. Pulse hard, and sufficiently full to demand a second bleeding, which was very much cupped and buffy. The pediluvium has several times been made use of, and still is had recourse to occasionally. From this, as well as from heat applied in any form, he has received much temporary relief.—℞. Potassæ nitratis, ℈ij. Syr. papav. albi, f. ʒi. Aquæ puræ, f. ℥v. Mft. mistura, cochliaria duo quartis horis sumenda.

I need not give the daily particulars any further, for the preceding symptoms were the leading features of the disease throughout; neither need I add, that the antiphlogistic regimen was strictly followed. Frequently-repeated blood-lettings, however, soon abated the inflammation, and the blood abstracted, up to the Friday following, amounted to nearly 100 ounces; when he was pronounced convalescent.

*Observations.*—It is a curious fact, that, during the incipient stage of this disease, (when no febrile symptoms are perceptible, and when you draw blood to relieve the distressing pain in the side,) that blood shall not be inflamed; yet the inflammatory diathesis does not fail to show itself in a short space of time afterwards, and that too to an alarming degree.

Cases of pleuritis are but comparatively rare to what preceding years have produced; and this may be fairly accounted for from the great mildness of the season. I am very well aware that there is nothing novel in this case; neither should I have sought a place for it in your Journal, had I not observed the following circumstance:—During the time the inflammation was at its acme, and of course when the greater part of the blood was drawn, it was accidentally, at one of these periods, received into two vessels. After they had stood a little time, it was observed that the portion drawn just before closing the orifice had more of a buffy and cupped appearance than that which had been before taken. That which escaped first from the vein having been allowed to rest, it was found that the crassamentum was natural, excepting on its surface a small quantity of buff, about the size of an eighteen-penny piece; but the incrustation was so abundant on the surface of the other, that the crassamentum was totally hidden from view. Now, this is a circumstance which I have never before observed; neither have I seen an attempt to account for it in any author which I have consulted. I have never yet heard it doubted that the blood, either in a diseased or healthy state, was any other than *homogeneous* throughout; yet this experiment, which was more than once repeated, would seem to argue the contrary. If, according to the theory of Dr. Fowler, inflammation consisted merely in *increased action alone*, its consistency and similarity should have been the same in every particular. If this had been the case, each portion of blood should have been proportionately cupped and buffed. I do not, however, mean to enter on the different theories of inflammation which have been advanced, lest I get into a labyrinth in which I might, with all imaginable ease, lose myself in idle speculations and useless hypotheses. I should, therefore, feel desirous of putting the question through the medium of your valuable Journal.

*Lostwithiel; December* 12, 1818.

